# Transcriptomics and Phenotyping Define Genetic Signatures Associated with Echinocandin Resistance in Candida auris

**DOI:** 10.1128/mbio.00799-22

**Published:** 2022-08-15

**Authors:** Sabrina Jenull, Raju Shivarathri, Irina Tsymala, Philipp Penninger, Phan-Canh Trinh, Filomena Nogueira, Manju Chauhan, Ashutosh Singh, Andriy Petryshyn, Anton Stoiber, Anuradha Chowdhary, Neeraj Chauhan, Karl Kuchler

**Affiliations:** a Department of Medical Biochemistry, Max Perutz Labs Vienna, Medical University of Vienna, Campus Vienna Biocenter, Vienna, Austria; b Functional Microbiology, Institute of Microbiology, Department of Pathobiology, University of Veterinary Medicine, Vienna, Austria; c Public Health Research Institute, New Jersey Medical School, Rutgers, The State University of New Jersey, Newark, New Jersey, USA; d Department of Microbiology, Biochemistry and Molecular Genetics, New Jersey Medical School, Rutgers, The State University of New Jersey, Newark, New Jersey, USA; e CCRI-St. Anna Children’s Cancer Research Institute, Vienna, Austria; f National Reference Laboratory for Antimicrobial Resistance in Fungal Pathogens, Medical Mycology Unit, Department of Microbiology, Vallabhbhai Patel Chest Institute, University of Delhi, Delhi, India; CDC

**Keywords:** *Candida auris*, antifungal resistance, phenotypic variation, transcriptomics

## Abstract

Candida auris emerged as a human fungal pathogen only during the past decade. Remarkably, C. auris displays high degrees of genomic diversity and phenotypic plasticity, with four major clades causing hospital outbreaks with high mortality and morbidity rates. C. auris can show clinical resistance to all classes of antifungal drugs, including echinocandins that are usually recommended as first-line therapies for invasive candidiasis. Here, we exploit transcriptomics coupled with phenotypic profiling to characterize a set of clinical C. auris isolates displaying pronounced echinocandin resistance (ECN-R). A hot spot mutation in the echinocandin *FKS1* target gene is present in all resistant isolates. Moreover, ECN-R strains share a core signature set of 362 genes differentially expressed in ECN-R isolates. Among others, mitochondrial gene expression and genes affecting cell wall function appear to be the most prominent, with the latter correlating well with enhanced adhesive traits, increased cell wall mannan content, and altered sensitivity to cell wall stress of ECN-R isolates. Moreover, ECN-R phenotypic signatures were also linked to pathogen recognition and interaction with immune cells. Hence, transcriptomics paired with phenotyping is a suitable tool to predict resistance and fitness traits as well as treatment outcomes in pathogen populations with complex phenotypic diversity.

## INTRODUCTION

The connection between the environment and human health is becoming increasingly clear. Climate change and human-imposed environmental destruction facilitate the emergence of new pathogens but also promote the development of antimicrobial drug resistance (1–3). The latter is further exacerbated by the extensive use and misuse of antimicrobials in agricultural, animal mass production, and clinical settings (4, 5). For instance, climate change may have promoted the emergence of the fungus Candida auris as a human pathogen, which was recently isolated from the natural marine environment in India (6, 7). C. auris was first identified in 2009 (8), and invasive infections were considered rare (9). However, over the last years, C. auris spread globally, causing outbreaks in health care facilities in more than 50 countries (10). Remarkably, C. auris emerged seemingly simultaneously at distinct geographical locations and is classified into four major phylogenetic clades (11). Furthermore, C. auris clades display substantial genetic diversity and show distinct phenotypic traits, especially diminished susceptibilities to antifungal therapies (11–14). For instance, the majority of all isolates, with the exception of clade II strains, are resistant to fluconazole (FCZ). Importantly, cross-resistance to drugs such as amphotericin B (AmB) and echinocandins (ECNs) is additionally occurring among isolates from distinct clades and geographical regions. Of note, numerous isolates displaying multidrug resistance (MDR) to two or all classes of antifungals have been recovered among clade I, clade III, and clade IV isolates (12, 13, 15, 16), with 41% of isolates displaying multidrug resistance and up to 4% displaying panresistance (10). Since ECN and AmB resistance can be rapidly acquired (17), the U.S. Centers for Disease Control and Prevention (CDC) highlighted C. auris as an urgent antibiotic resistance threat (18). Furthermore, diagnostic tools tailored to routine detection are scarce (19), and C. auris shows pronounced adhesion to abiotic and biotic surfaces such as human skin, thus facilitating human-to-human transmission in clinical settings (20).

The fungal cell wall plays pivotal roles in maintaining cellular integrity under normal as well as extreme environmental conditions (21). The cell wall architecture comprises the innermost chitin layer subtending glucan layers, with mannosylated proteins forming the outermost layers (22, 23). The fungicidal action of ECN is based on its inhibition of the *FKS* 1,3-β-glucan synthase genes. Indeed, *FKS1* point mutations are commonly detected in ECN-resistant (ECN-R) *Candida* spp. (24, 25). Moreover, the cell wall, the first point of contact with the host (26), holds an array of pathogen-associated molecular patterns (PAMPs) engaging with host pattern recognition receptors (PRRs) that mediate pathogen recognition and antifungal effector mechanisms such as phagocytosis and the release of cytotoxic reactive oxygen species (ROS) (27). The structural features of cell wall mannose units vary among C. auris isolates, which is most likely affecting inflammatory host responses (28). In addition, host interaction or colonization abilities differ among C. auris clades (28, 29), underpinning the complex population structure of C. auris. However, the molecular mechanisms that facilitate the distinct biological properties of C. auris isolates remain elusive. While pioneering studies provided important insights into genomic plasticity (11, 13, 14), complementary studies are needed to explain the complex population structures driven by mechanisms beyond genomic diversity. Recently, studies exploiting transcriptomics, proteomics, and metabolomics have offered new insights into the molecular signatures of C. auris phenotypic diversity. For instance, transcriptional profiling of C. auris cells growing as cell aggregates revealed the increased transcription of certain cell wall-related genes compared to C. auris cells with nonaggregating phenotypes (30). Another comparative study explored the metabolomic, lipidomic, and proteomic differences of two C. auris isolates with distinct antifungal susceptibilities compared with Candida albicans (31). Of note, the extracellular vesicle contents of different isolates suggest distinct immunomodulatory properties (32). Recent work from our group demonstrated that specific transcriptional signatures correlate with phenotypic traits of C. auris patient isolates with opposing antifungal susceptibility profiles (33). Therefore, we further aimed to uncover additional signatures among clade I C. auris isolates with distinct phenotypic traits. Antifungal susceptibility profiling of clinical isolates identified a cluster of three ECN-R isolates, which were further compared to two other unrelated ECN-susceptible (ECN-S) strains using RNA sequencing (RNA-seq). Although all isolates are of clade I, large transcriptional alterations exist between ECN-R and ECN-S isolates under basal growth conditions, as a set of 362 genes was differentially expressed in all ECN-R isolates. These transcriptional profiles suggest distinct cell surface properties, reflected in the increased adhesion and enhanced host recognition of ECN-R isolates. Our work shows the utility of transcriptomic signatures to predict the phenotypic diversity of C. auris isolates and suggests that biological properties related to C. auris virulence may be reflected in certain subcluster traits.

## RESULTS

### Characterization of C. auris patient isolates.

**FIG 1:**
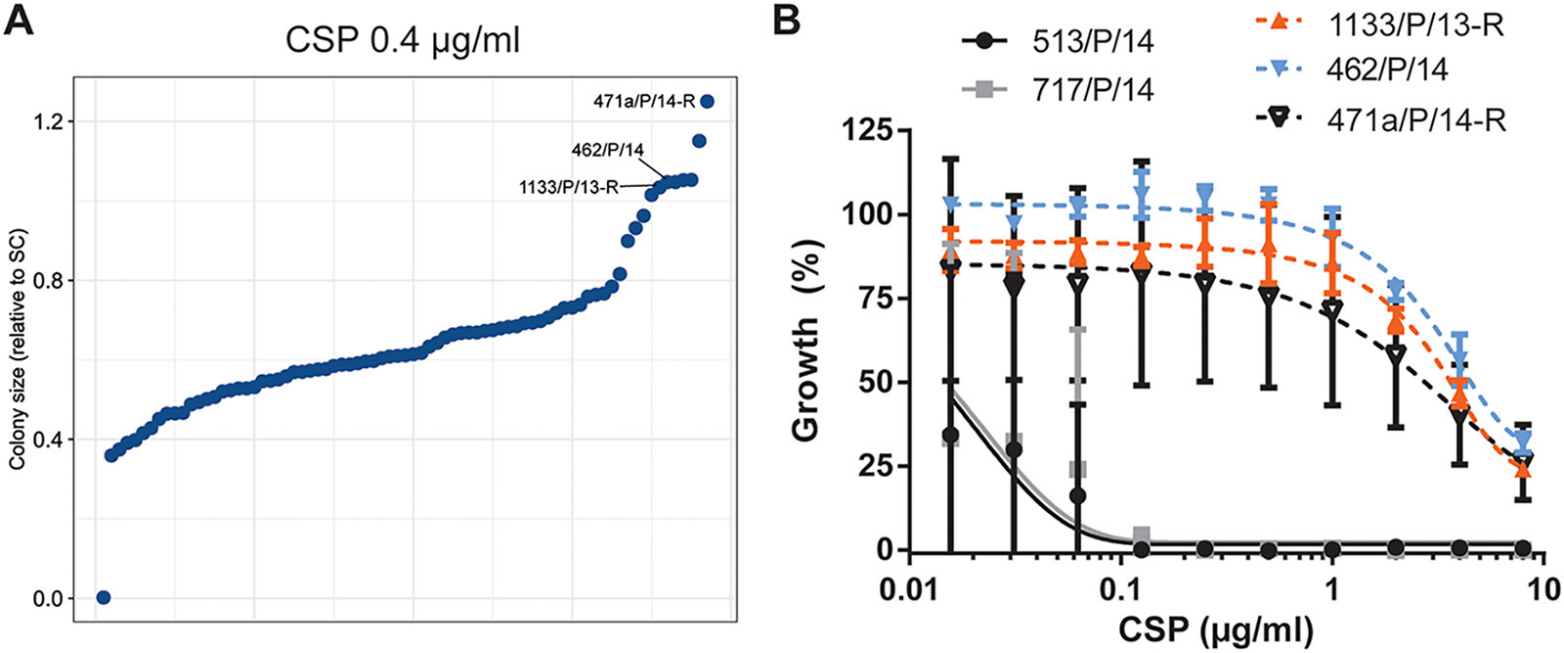
Identification of caspofungin-resistant C. auris patient isolates. (A) Antifungal susceptibility screen on solid medium of clade I C. auris patient isolates. Cultures grown overnight in YPD medium at 30°C were spotted onto synthetic complete medium plates supplemented with caspofungin (CSP). Colony growth was imaged after 3 days of incubation at 30°C and used to assess the relative colony size (ratio of the colony size with an antifungal to that with no antifungal). The relative colony size was used to rank (CSP) susceptibilities among the tested isolates. Values represent the means from 3 biological replicates. (B) Confirmation of plate-based screening results using liquid growth inhibition assays. Cells were incubated in YPD medium with CSP at the indicated concentrations at 30°C for 24 h prior to OD_600_ measurements. Data represent the means ± SD from 3 biological replicates. (See also Fig. S1 in the supplemental material.)

The antifungal susceptibilities and MICs of C. auris can differ strikingly at both the inter- and intraclade levels (12, 14). Therefore, an automated antifungal testing pipeline can be useful for prescreening a larger set of clinical isolates to establish comparative antifungal susceptibility profiles. Recently, we analyzed the antifungal susceptibilities of 21 C. auris patient isolates belonging to clade I. We detected various antifungal susceptibility profiles (33), as also reported previously by others (12, 13). We included 64 additional clade I C. auris patient isolates and analyzed their susceptibility profiles on solid synthetic complete (SC) agar medium against antifungals representing all four major antifungal classes (see Materials and Methods; see also Fig. S1A to C in the supplemental material). The majority of clade I isolates displayed limited inhibition by FCZ and various susceptibilities to AmB (12). ECNs are recommended as first-line antifungal therapies against C. auris (34). Although resistance to ECN has been considered infrequent (35), recent reports suggest an ECN resistance rate of between 2 and 7% among C. auris isolates (12). Our screen identified isolates with limited caspofungin (CSP) susceptibilities, with isolate 471a/P/14-R being the least susceptible among the tested patient isolates (Fig. 1A). Moreover, we found two additional isolates that clustered with 471a/P/14-R based on the colony size reachable upon antifungal treatment (Fig. S1A, black box): 1133/P/13-R and 462/P/14. Like 471a/P/14-R, these isolates displayed low CSP susceptibility (Fig. 1A). Importantly, limited ECN susceptibility was reported for all 3 isolates in a previous study (36), thus confirming the robustness of our phenotyping. To delineate the conserved mechanisms mediating decreased CSP efficacy, we set out to further characterize these isolates in comparison to CSP-susceptible isolates. We chose isolates 513/P/14 and 717/P/14 as they displayed otherwise similar antifungal susceptibility profiles (Fig. S1A, marked with asterisks). Since the above-described solid-medium robotic screen is not a validated or clinically approved method for antifungal susceptibility testing, we confirmed the decreased growth inhibition upon CSP treatment of isolates 471a/P/14-R, 1133/P/13-R, and 462/P/14 in liquid yeast extract-peptone-dextrose (YPD) medium in comparison to the susceptible isolate controls 513/P/14 and 717/P/14. Of note, all five isolates displayed comparable AmB and FCZ susceptibilities (Fig. S1D and E). In addition, MIC assays according to Clinical and Laboratory Standards Institute (CLSI) protocols further verified the low CSP susceptibilities of isolates 471a/P/14-R, 1133/P/13-R, and 462/P/14 (Table 1). Additionally, other ECNs such as micafungin (MFG) and anidulafungin (AFG) exerted limited growth inhibition against 471a/P/14-R, 1133/P/13-R, and 462/P/14. Based on CDC-suggested breakpoints for ECNs (MICs of >4 μg/mL for AFG and MFG and >2 μg/mL for CSP [37]), these isolates can be considered ECN-R, as pointed out previously (36).

**TABLE 1:** MIC values as determined by the CSLI broth microdilution method^*a*^

Isolate	Source	Hospital/yr of isolation	MIC (μg/mL)									
			ICZ	VCZ	ISZ	PSZ	AmB	CSP	MFG	AFG	FCZ	5FC
1133/P/13-R	Blood	Hosp1/2013	0.5	2	0.5	0.25	8	8	8	8	64	0.5
462/P/14	Blood	Hosp2/2014	0.125	1	0.25	0.015	0.5	4	8	8	64	0.5
471a/P/14-R	Blood	Hosp2/2014	0.25	1	0.125	0.015	0.5	8	8	8	64	0.5
513/P/14	Blood	Hosp3/2014	2	4	2	1	4	1	0.25	0.5	64	0.25
717/P/14	Tissue	Hosp1/2014	0.125	2	0.125	0.015	4	0.25	0.125	0.125	64	32

a ICZ, itraconazole; VCZ, voriconazole; ISZ, isavuconazole; PSZ, posaconazole; AmB, amphotericin B; CSP, caspofungin; MFG, micafungin; AFG, anidulafungin; FCZ, fluconazole; 5FC, 5-flucytosine.

10.1128/mbio.00799-22.1 FIG S1 Antifungal susceptibilities of C. auris patient isolates (related to Fig. 1). (A) Hierarchical clustering and heatmap visualization of the antifungal susceptibility screen described in the legend of Fig. 1A. The black box represents a cluster of isolates with low CSP susceptibility. Rows marked with asterisks highlight isolates used as CSP-susceptible controls in follow-up experiments. (B and C) Ranked colony size ratios from antifungal susceptibility screening in panel A for fluconazole (FCZ) (B) and amphotericin B (AmB) (C). Mean values from 3 biological replicates are plotted, and isolates chosen for further investigation are highlighted. (D and E) Growth inhibition assay in liquid YPD medium in response to FCZ (D) and AmB (E) as described in the legend of Fig. 1B. Mean values and SD from 3 biological replicates are shown. Download FIG S1, TIF file, 2.1 MB. Copyright © 2022 Jenull et al. 2022 Jenull et al. https://creativecommons.org/licenses/by/4.0/ This content is distributed under the terms of the Creative Commons Attribution 4.0 International license.

### ECN-R isolates display distinct transcriptomic profiles.

**FIG 2:**
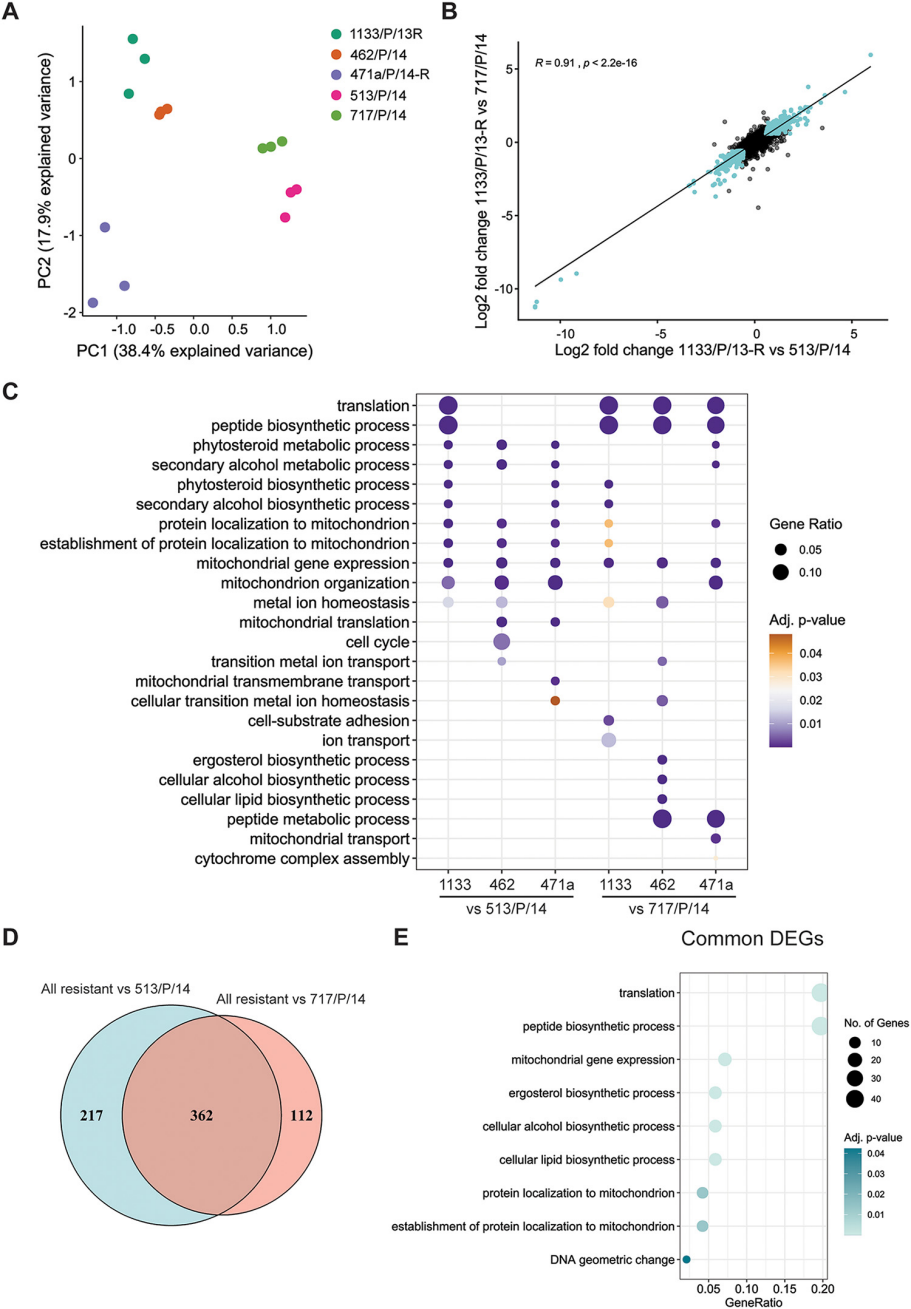
Transcriptional profiles of ECN-R and ECN-S patient isolates. (A) Principal-component analysis (PCA) based on normalized RNA-seq read counts (counts per million [CPM]). (B) Scatterplot depicting the log_2_ fold changes in transcript abundances for the indicated pairwise comparisons. The Pearson correlation coefficient (*R*) and linear regression line are indicated. Turquoise dots represent common differentially expressed genes (DEGs) (FC of >1.5; FDR of <0.05) in both comparisons. (C) GO enrichment analysis of DEGs (FC of >1.5; FDR of <0.05) for the indicated comparisons. The dot size represents the gene ratio, meaning the number of genes enriched in the plotted GO term relative to the total number of DEGs used as the input data. Due to space reasons, 1133/P/13-R, 462/P/14, and 471a/P/14-R are abbreviated 1133, 462, and 471a, respectively. (D) Venn diagram depicting the overlap of common genes differentially expressed (FDR of <0.05; FC of >1.5) between all ECN-R isolates (471a/P/14-R, 1133/P/13-R, and 462/P/14 [“All resistant”]) and ECN-S isolates 513/P/14 and 717/P/14. (E) GO enrichment analysis of common DEGs between all ECN-R isolates and both ECN-S isolates (Venn diagram intersection in panel D). The gene ratio represents the number of genes enriched in the plotted GO term relative to the total number of DEGs used as the input data. The dot size reflects the number of genes enriched in the corresponding GO term. (See also Fig. S2 in the supplemental material.)

To better understand whether ECN-R isolates share additional characteristics that make them distinguishable from ECN-S isolates, we further characterized these isolates at the molecular level. Therefore, we performed comparative transcriptomics analyses of ECN-R and ECN-S isolates grown in liquid YPD medium at 30°C. A principal-component analysis (PCA) of normalized read counts demonstrated that ECN-R (471a/P/14-R, 1133/P/13-R, and 462/P/14) and ECN-S (513/P/14 and 717/P/14) isolates display distinct transcriptomes (Fig. 2A) (PC1, 38% variance). This was further reflected by the high number of differentially expressed genes (DEGs), which affected 24 to 12% of all detected C. auris coding sequences (CDSs) being at least 1.5-fold up- or downregulated in ECN-R compared to ECN-S isolates (Table 2). Interestingly, only 93 genes, representing 2% of C. auris CDSs (detected by RNA-seq here), were deregulated between two independent ECN-S isolates, 513/P/14 and 717/P/14 (Table 2). In comparison, 7% of detected CDSs were differentially expressed between the ECN-R isolates 462/P/14 and 471a/P/14-R (Table 2), with the latter forming a subcluster among ECN-R isolates (Fig. 2A). Yet the total number of DEGs is more than 3-fold smaller when 471a/P/14-R is compared to ECN-S isolates. For instance, 1,192 and 1,284 genes were differentially expressed in 471a/P/14-R with respect to the ECN-S 717/P/14 and 513/P/14 controls, respectively. In comparison, altered expression of 356 genes was detected between ECN-R isolates 471a/P/14-R and 462/P/14 (Table 2). As we observed a substantial number of DEGs, we further assessed whether the direction of transcriptional deregulation (up- or downregulated) is conserved in ECN-R isolates compared to both ECN-S isolates. Therefore, we directly compared the fold changes (FCs) in gene expression between one ECN-R isolate and both ECN-S strains (i.e., 1133/P/13-R versus 513/P/14 and 1133/P/13-R versus 717/P/14), demonstrating a high correlation of deregulated genes (*R* ≥ 0.88) (Fig. 2B and Fig. S2A and B). Gene ontology (GO) analysis of DEGs (false discovery rate [FDR] of <0.05; >1.5-fold change) further revealed that related biological processes are commonly deregulated in ECN-R isolates compared to each ECN-S C. auris isolate (Fig. 2C and Table S4). For instance, genes associated with mitochondrial gene expression (adjusted *P* value of 4.49 × 10^−05^) were commonly enriched in all resistant versus susceptible strains (Fig. 2C), showing predominantly enhanced expression in ECN-R strains (Fig. S2C). Accordingly, 579 or 474 common genes were differentially regulated among all ECN-R isolates compared to ECN-S isolate 513/P/14 or 717/P/14, respectively (Fig. S2D and E). These common DEGs represent 34% and 30% of all DEGs from ECN-R-versus-513/P14 (579 out of 1,702) and ECN-R-versus-717/P/14 (474 out of 1,559) comparisons, respectively. Collectively, the related transcriptomic alterations and similar biological processes affected between ECN-R and ECN-S isolates, as well as the substantial overlap of DEGs, suggest a core signature gene set linked to ECN-R traits in unrelated isolates. Therefore, we next aimed to define the core set of common DEGs between ECN-R and ECN-S C. auris isolates. We identified a set of 362 DEGs shared among all three ECN-R isolates, compared to both ECN-S isolates (Fig. 2D), and denoted them ECN-R core genes. Functionally, core genes were linked to three major biological functions, including translation (adjusted *P* value of 4.91 × 10^−09^), ergosterol biosynthetic processes (adjusted *P* value of 4.91 × 10^−09^), and mitochondrial gene expression (adjusted *P* value of 1.12^−07^), as revealed by GO enrichment analysis (Fig. 2E and Table S4). Genes linked to mitochondrial gene expression and function included mitochondrial translation initiation and elongation factors (*IFM1* and *TUF1*, respectively) as well as several mitochondrial ribosomal proteins (e.g., *RSM4*, *MRPL36*, B9J08_000156, and B9J08_000569) (Table S4; see also Table S3 for C. auris gene identifiers). Additionally, genes implicated in mitochondrial protein import (*TOM70*, *TOM40*, *TOM22*, *TIM50*, *TIM44*, *TIM40*, and *TIM23*) were upregulated in all ECN-R isolates (see Table S3 for C. auris gene identifiers), suggesting altered mitochondrial function in ECN-R isolates. Therefore, we tested the susceptibility of ECN-R strains to the respiratory chain inhibitor antimycin A (38). Indeed, the ECN-R isolates displayed roughly 2- to 3-fold-decreased antimycin A 50% inhibitory concentration (IC_50_) values compared to the ECN-S isolates (Fig. S2F). In summary, these data demonstrate that ECN-R isolates show distinct transcriptional profiles, affecting about 6% of all C. auris genes (362 out of 5,397 C. auris genes detected here).

**TABLE 2:** Differentially expressed genes in comparisons of ECN-R versus ECN-S isolates^*a*^

Comparison	Resistance	No. of genes			
		Upregulated	Downregulated	Total	% of total genes
1133/P/13-R vs 513/P/14	ECN-R vs ECN-S	645	497	1,142	21
462/P/14 vs 513/P/14	ECN-R vs ECN-S	496	308	804	15
471a/P/14-R vs 513/P/14	ECN-R vs ECN-S	755	529	1,284	24
1133/P/13-R vs 717/P/14	ECN-R vs ECN-S	507	452	959	18
462/P/14 vs 717/P/14	ECN-R vs ECN-S	368	305	673	12
471a/P/14-R vs 717/P/14	ECN-R vs ECN-S	668	524	1,192	22
513/P/14 vs 717/P/14	ECN-S vs ECN-S	12	81	93	2
462/P/14 vs 471a/P/14-R	ECN-R vs ECN-R	190	166	356	7

a The ECN-R isolates were 1133/P/13-R, 462/P/14, and 471a/P/14-R. The ECN-S isolates were 513/P/14 and 717/P/14. The cutoffs were an FDR of <0.05 and a log_2_ fold change of >0.58.

10.1128/mbio.00799-22.2 FIG S2 Genes differentially regulated in ECN-R patient isolates (related to Fig. 2). (A and B) Scatterplot depicting the log_2_ fold changes in transcript abundances for the indicated comparisons. Pearson correlation coefficients (*R*) and linear regression lines are indicated. Turquoise dots represent common genes differentially expressed (FC of >1.5; FDR of <0.05) in both comparisons. (C) Heatmap depicting the log_2_ fold changes of genes associated with mitochondrial gene expression for the indicated pairwise comparisons. Gene names refer to C. albicans homologues. C. auris gene identifiers are depicted if no gene name was annotated to the C. albicans homologue. Due to space reasons, 1133/P/13-R, 462/P/14, and 471a/P/14-R are abbreviated 1133, 462, and 471a, respectively. Table S5 in the supplemental material shows the gene list and the corresponding identifiers. (D and E). Venn diagram depicting the overlap of common DEGs between ECN-R isolates and ECN-S isolate 513/P/14 (D) or ECN-S isolate 717/P/14 (E). *n* depicts the total number of regulated genes. (F) Growth inhibition assay with antimycin A. Cells were incubated in YPD medium with the indicated concentrations of antimycin A at 30°C for 24 h prior to OD_600_ measurements. IC_50_ values were calculated by using a nonlinear regression (four-parameter dose-response curve) model. Mean values plus SD (line graph) or 95% confidence intervals (CI) (IC_50_ table) from 4 to 5 biological replicates are depicted. Download FIG S2, TIF file, 2.5 MB. Copyright © 2022 Jenull et al. 2022 Jenull et al. https://creativecommons.org/licenses/by/4.0/ This content is distributed under the terms of the Creative Commons Attribution 4.0 International license.

10.1128/mbio.00799-22.7 TABLE S3 Differential gene expression analysis results (related to Fig. 2). Download Table S3, XLSX file, 1.5 MB. Copyright © 2022 Jenull et al. 2022 Jenull et al. https://creativecommons.org/licenses/by/4.0/ This content is distributed under the terms of the Creative Commons Attribution 4.0 International license.

**FIG 3:**
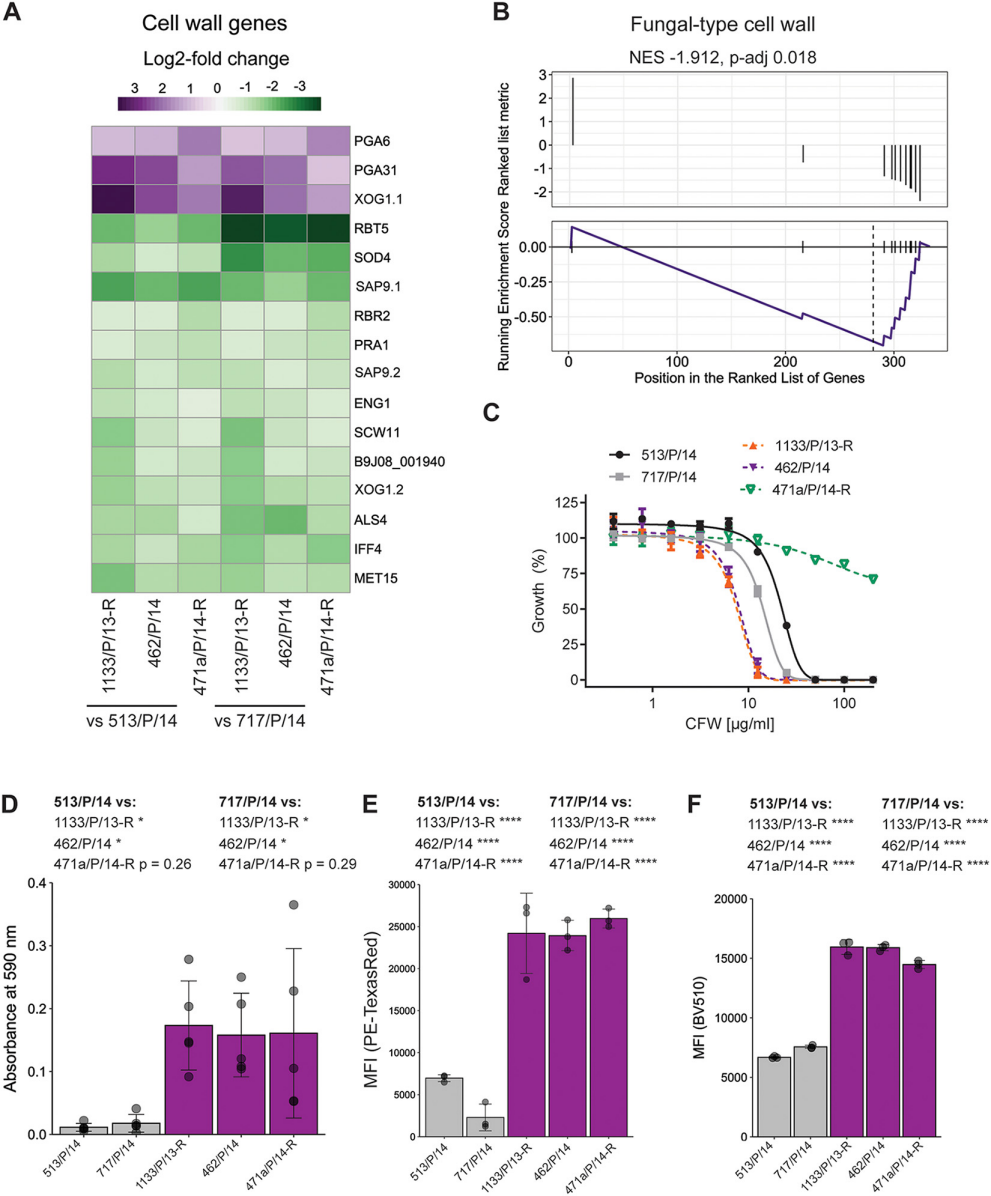
ECN-R isolates show altered cell surface properties. (A) Heatmap depicting log_2_ fold changes of genes that contain “cell wall” in the fungal-type cellular component GO term category and that are commonly differentially expressed (FDR of <0.05; FC of >1.5) between all ECN-R isolates and both ECN-S C. auris isolates. Gene names refer to C. albicans homologues. Notably, two C. auris genes were found to be homologous to C. albicans *XOG1* and *SAP9*. Those genes are depicted as *XOG1*.*1* and *XOG1.2* and *SAP9.1* and *SAP9.2*, respectively. C. auris gene identifiers are depicted if no gene name was annotated to the C. albicans homologue. Table S5 in the supplemental material presents the gene list and the corresponding identifiers. (B) Commonly differentially expressed genes between ECN-R and ECN-S isolates were ranked according to their log_2_ fold changes between 1133/P/13-R and 513/P/14 and subjected to gene set enrichment analysis (GSEA). The plot depicts the ranked gene list (top) and the enrichment score (bottom) for the GO term category “fungal-type cell wall.” (C) Growth inhibition in liquid YPD medium in response to calcofluor white (CFW) at the indicated concentrations. OD_600_ values were measured after 24 h at 30°C. The percentage of growth upon CFW treatment relative to growth in YPD medium is depicted. Data represent the means and SD from three biological replicates. (D) Adhesion to plastic as assessed by crystal violet staining after 4 h of static YPD culture in polystyrene plates at 37°C. Mean values and SD from 5 biological replicates are plotted. (E) Concanavalin A-Texas Red staining of C. auris cells grown to the exponential growth phase in YPD medium at 30°C. The mean fluorescence intensity (MFI) is depicted. Data represent the means and SD from 3 biological replicates. (F) Aniline blue staining of C. auris cells grown as described above for panel E. The MFI is depicted, and data represent the means plus SD from 3 biological replicates. *, *P* < 0.05; ****, *P* < 0.00001 (by one-way ANOVA and Tukey’s multiple-comparison test for the indicated comparisons). Statistical homoscedasticity of the data was assessed by Bartlett’s test. The data presented in panel D were heteroscedastic, and hence, Welch’s ANOVA was applied. NES, normalized enrichment score. (See also Fig. S3.)

10.1128/mbio.00799-22.8 TABLE S4 Gene ontology (GO) analysis and gene set enrichment analysis (GSEA) results (related to Fig. 2 and 3). Download Table S4, XLSX file, 0.03 MB. Copyright © 2022 Jenull et al. 2022 Jenull et al. https://creativecommons.org/licenses/by/4.0/ This content is distributed under the terms of the Creative Commons Attribution 4.0 International license.

### ECN-R isolates have altered cell surface properties.

The mechanisms and modulators of ECN susceptibility have been explored in other *Candida* spp. such as C. albicans and C. glabrata (39–44). To assess whether genes previously implicated in CSP susceptibility in C. albicans are also regulated in C. auris ECN-R isolates, we retrieved the relevant genes from the *Candida* Genome Database (CGD) (http://www.candidagenome.org/), which contained 110 genes. We then assessed the expression of the corresponding C. auris homologues in ECN-R versus ECN-S isolates. For instance, we found 20 genes differentially regulated in ECN-R isolate 1133/P/13-R compared to ECN-S isolate 513/P/14 (Fig. 3B). Of note, the ECN targets *FKS1* and *FKS2* were not differentially regulated at the mRNA level (Fig. S3A). However, three genes known to modulate CSP susceptibility in C. albicans were commonly deregulated in all ECN-R C. auris isolates compared to both ECN-S isolates (Fig. S3C). These genes included the transcription factor *NRG1* (B9J08_005429), the kinase *GIN4* (B9J08_005249), and the cell wall-associated gene *PGA31* (B9J08_000117) (Fig. S3C). As alterations in the cell wall architecture impact CSP susceptibility (45), we further inspected genes related to cell wall function (GO cellular component annotation containing “cell wall”) among the core set in ECN-R isolates. Thereby, we identified 16, predominantly downregulated, genes in ECN-R isolates (Fig. 3A). Similarly, gene set enrichment analysis (GSEA) revealed the enrichment of fungal-type cell wall genes among the differentially expressed ECN-R core genes (Fig. 3B). Among the regulated cell wall genes were homologues of C. albicans glucanase genes (*XOG1* and *ENG1*) (46, 47) as well as genes encoding putative adhesins (*ALS4*, *IFF4*, and *PGA6*) (48–50). These data suggest that ECN-R isolates harbor altered cell wall structure and surface properties. Indeed, ECN-R C. auris isolates showed enhanced adhesion to plastic and differential susceptibilities to the cell wall-perturbing agent calcofluor white (CFW) (Fig. 3C and D). Notably, adhesive properties were variable for isolate 471a/P/14-R, which also showed decreased sensitivity to CFW, while 1133/P/13-R and 462/P/14 were more susceptible to CFW treatment than the ECN-S isolates (Fig. 3C). Finally, we confirmed the differences in cell wall composition between ECN-S and ECN-R isolates, with the latter showing increased mannan and β-glucan contents (Fig. 3E and F). Notably, the exposure of β-glucans was slightly decreased compared to that of the susceptible control isolate 513/P/14. However, the same trend was also seen when 513/P/14 was compared to ECN-S isolate 717/P/14. The chitin abundance was unaltered among the isolates (Fig. S3E).

10.1128/mbio.00799-22.3 FIG S3 ECN-R isolates harbor altered cell surface properties (related to Fig. 3). (A) Scatterplot depicting the log counts per million reads (CPM) and the log_2_ fold changes (log_2_FC) in transcript abundances between 1133/P/13-R and 513/P/14. C. albicans homologues previously shown to affect caspofungin susceptibility in C. albicans are highlighted in yellow, and the ECN targets *FSK1* and *FKS2* are represented in green. Dotted lines represent log_2_ fold change values of 0.58 and −0.58. (B) Heatmap of “caspofungin-phenotype” (as in panel A) genes depicting log_2_ fold changes in transcript abundances for the indicated comparisons. Genes based on differential expression (FDR of <0.05; FC of >1.5) in 1133/P/13-R versus 513/P/14 are shown. Genes commonly differentially expressed in all comparisons are highlighted with asterisks. C. auris gene identifiers are depicted if no gene name was annotated to the C. albicans homologue. See Table S5 in the supplemental material for the full analysis results. (C) Gating strategy for flow cytometry analysis of the cell wall components shown in panel D and Fig. 3E. (D) Dectin-1 anti-Fc-FITC and calcofluor white staining to detect exposed β-glucan and total chitin, respectively, in cells grown to the exponential growth phase in YPD medium at 30°C. Mean fluorescence intensity (MFI) values of single cells (singlet populations depicted in panel D) are shown. Data represent the mean values and SD from biological quadruplicates. (E) Adhesion to plastic, assessed as described in the legend of Fig. 3D, of the indicated isolates and gene deletion derivatives. Mean values and SD from 3 biological replicates are plotted. (F) Assay of growth inhibition by caspofungin for the indicated isolates and the *pga6*Δ derivative in liquid YPD medium after 24 h at 30°C. *, *P* < 0.05; **, *P* < 0.01 (by Welch’s ANOVA followed by Tukey’s multiple-comparison correction). The statistical homoscedasticity of the data was tested by performing Bartlett’s test. The data presented in panel D were heteroscedastic, and hence, Welch’s ANOVA was applied. Download FIG S3, TIF file, 2.3 MB. Copyright © 2022 Jenull et al. 2022 Jenull et al. https://creativecommons.org/licenses/by/4.0/ This content is distributed under the terms of the Creative Commons Attribution 4.0 International license.

10.1128/mbio.00799-22.9 TABLE S5 List of genes depicted in the indicated heatmaps (related to Fig. 3 and Fig. S2 and S3). Download Table S5, XLSX file, 0.03 MB. Copyright © 2022 Jenull et al. 2022 Jenull et al. https://creativecommons.org/licenses/by/4.0/ This content is distributed under the terms of the Creative Commons Attribution 4.0 International license.

The fungal cell wall is a dynamic structure that is affected by environmental conditions (51) and alterations in the abundances of cell wall-modulating enzymes (52, 53) and mannosylated proteins (54). *PGA6* (B9J08_001366), a homologue of a putative C. albicans glycosylphosphatidylinositol (GPI)-anchored adhesion molecule (49), was among the upregulated cell wall-related genes in ECN-R isolates, in addition to Putative GPI-anchored protein (*PGA31*) and the exoglucanase gene *XOG1* (46) (Fig. 3A). Interestingly, the ectopic overexpression or deletion of other PGA family proteins affects fungal adhesion as well as CSP sensitivity (45, 54), and *PGA6* was found to be upregulated in CSP-treated C. auris cells (55). Therefore, we constructed a deletion mutant lacking *PGA6* to test whether it contributes to ECN-R and adhesion in C. auris. However, the loss of *PGA6* in ECN-R isolates 471a/P/14-R, 1133/P/13-R, and 462/P/14 only moderately reduced adhesion (Fig. S3E) and did not affect CSP susceptibility (Fig. S3F).

### ECN-R isolates trigger enhanced macrophage responses.

**FIG 4:**
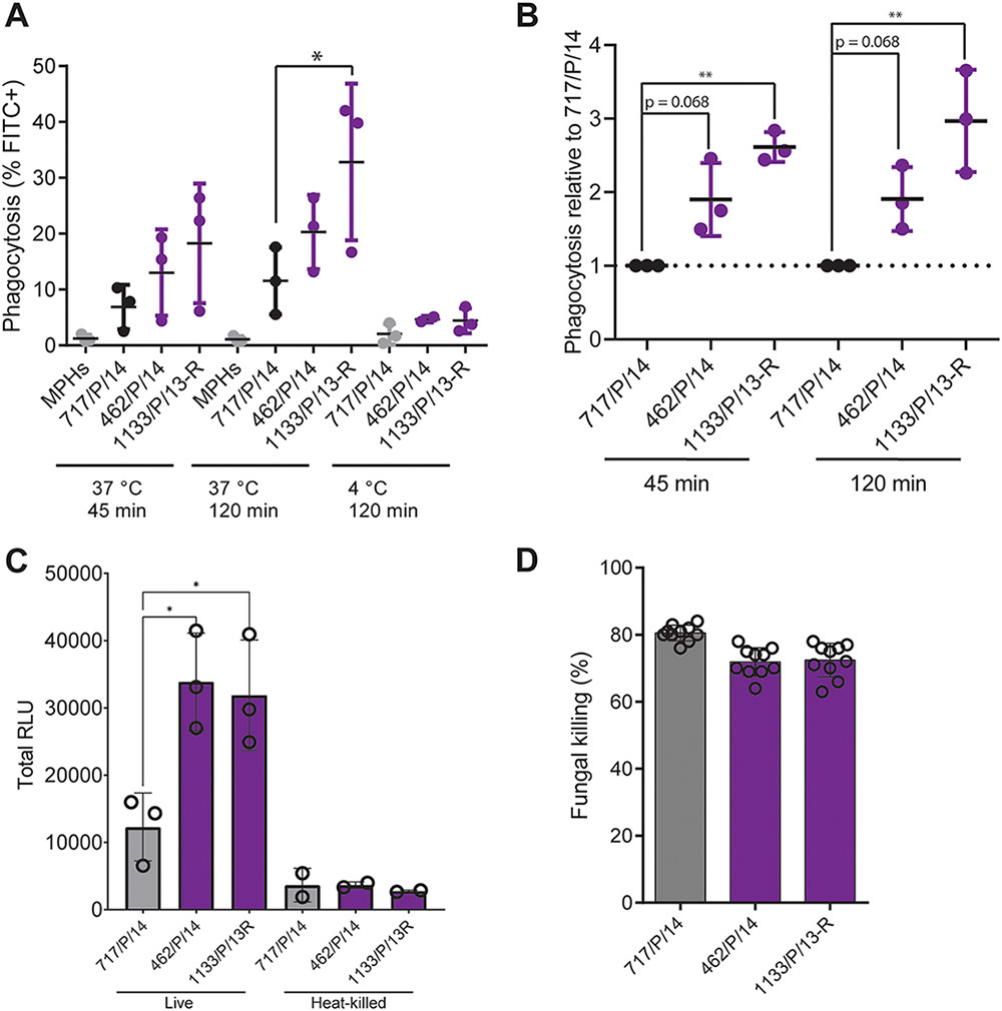
ECN-R isolates undergo distinct recognition by macrophages. (A and B) Phagocytosis of FITC-labeled C. auris cells by bone marrow-derived macrophages (BMDMs) after 45 and 120 min of coculture as assessed by flow cytometry. Macrophages were gated according to the scheme shown in Fig. S4A in the supplemental material, and the percentages of FITC-positive (FITC^+^) macrophages (A) and relative phagocytosis (relative to mean 717/P/14 levels) (B) are depicted. The means (horizontal lines) and SD from biological triplicates (dots) are shown. (C) Real-time luminescence-based reactive oxygen species (ROS) assay with BMDMs cocultured with live or heat-killed (70°C for 10 min) C. auris cells. Relative luminescence units (RLU) were recorded every 2.5 min for at least 130 min. Representative RLU over time per 1,000 BMDMs are depicted in Fig. S4B. Data represent the mean total RLU and SD from three biological replicates measured in technical triplicates. (D) Fungal survival after 4 h of coculture with macrophages. The percentage of fungal killing represents the number of CFU recovered after BMDM interaction relative to that in C. auris single cultures under otherwise identical conditions. Data represent the means and SD from two independent experiments performed with five technical replicates. n.s. (not significant), *P* > 0.05; *, *P* < 0.05; **, *P* < 0.01 (by one-way ANOVA followed by Tukey’s multiple-comparison correction). The homoscedasticity of the data was confirmed by performing Bartlett’s test. (See also Fig. S4.)

In addition to glucans, mannans are major drivers of host responses to C. auris (28). Since we detected major mannan increases in ECN-R isolates, we next assessed whether this affects host interactions with primary murine bone marrow-derived macrophages (BMDMs). We chose isolate 717/P/14 as a representative of ECN-S isolates and the ECN-R isolates 1133/P/13-R and 462/P/14. Fungal coculture experiments showed that the rate of phagocytosis of fluorescein isothiocyanate (FITC)-labeled ECN-R isolates was approximately 2-fold higher for 717/P/14 after 45-min and 120-min interactions with BMDMs (Fig. 4A and B). In addition, the release of reactive oxygen species (ROS) was diminished upon challenge with ECN-S isolate 717/P/14 compared to the responses to ECN-R isolates (Fig. 4C and Fig. S4C). However, killing by BMDMs was slightly enhanced for 717/P/14 cells after 4 h of coculture (Fig. 4D). Notably, ECN-S isolate 717/P/14 displayed an elevated growth rate under BMDM culture conditions compared to ECN-R isolates 1133/P/13-R and 462/P/14 (Fig. S4B). However, the fungal viability in cocultures with BMDMs was calculated relative to that of C. auris cultured without immune cells (see Materials and Methods) to minimize bias due to differential fungal growth under BMDM culture conditions. Collectively, these data demonstrate distinct phenotypic, transcriptomic, and antifungal host responses of ECN-R and ECN-S C. auris isolates. ECN-R traits are linked to a shared set of commonly dysregulated genes in C. auris isolates, suggesting that such transcriptomic fingerprints could be helpful for predicting antifungal resistance and virulence-related traits in C. auris.

10.1128/mbio.00799-22.4 FIG S4 Macrophage interaction with ECN-R and ECN-S C. auris isolates (related to Fig. 4). (A) Gating strategy to assess the phagocytosis rate (percent) of the FITC-labeled C. auris cells shown in Fig. 4A. (B) Luminescence-based reactive oxygen species (ROS) assay of macrophages cocultured with the indicated C. auris isolates. Relative luminescence units (RLU) per minute and 1,000 macrophages over time are plotted. Data show mean values and SD from technical triplicates from one representative biological replicate. The total RLU over time from biological replicates are depicted in Fig. 4C. (C) Growth of the indicated C. auris isolates under macrophage coculture conditions (L-conditioned DMEM with 10% hiFCS at 37°C with 5% CO_2_ under static conditions using a tissue culture-treated polystyrene plate). The data represent the means and SD from biological triplicates. Download FIG S4, TIF file, 1.8 MB. Copyright © 2022 Jenull et al. 2022 Jenull et al. https://creativecommons.org/licenses/by/4.0/ This content is distributed under the terms of the Creative Commons Attribution 4.0 International license.

## DISCUSSION

In addition to environmental stress, pathogens can face severe challenges by host immune defenses or therapeutic intervention strategies such as antibiotic or antifungal treatment (56). Phenotypic plasticity facilitating stress adaptation is therefore key for microbial survival. Multiple mechanisms enable adaptive traits, including genomic alterations or epigenetic modifications that govern transcriptional and phenotypic adaptation (57). The evolutionary diversification of C. auris and its emergence as a human pathogen are still poorly understood (58). Molecular clock analyses estimate that the last common ancestor for each C. auris clade arose within the last 360 years. In contrast, simultaneous clade diversification may have happened more recently (13). Of note, “clade-specific” environmental stresses coupled with anthropogenic actions may have been additional drivers (13, 59). This is reflected in the genetic and phenotypic diversities among C. auris clades (14). For instance, clade II isolates show a propensity for ear infections (60); they lost genes encoding putative adhesins (61) and harbor impaired stress resistance (62). Additionally, despite rather low genetic diversities within a clade (14), phenotypic intraclade variations, such as in antifungal susceptibility profiles (13, 33), cell wall structures (28), or aggregate formation (63), are known. Here, we further explored the intraclade diversity of clade I C. auris clinical isolates. We reveal pronounced transcriptional alterations affecting up to 24% of the C. auris genes detected here when ECN-S and ECN-R strains were compared. Remarkably, the deregulation of genes is reduced (2% of the detected C. auris genes) between independent and unrelated ECN-S clinical patient isolates. Interestingly, all tested isolates displayed FCZ resistance according to CLSI broth microdilution testing, while susceptibilities to AmB and 5-flucytosine varied among the isolates. Hence, transcriptomic alterations between these isolates may also reflect potential complex genetic compensatory mechanisms leading to distinct resistance traits that are not coupled solely to ECN-R. For instance, cell wall alterations have also been found in Candida tropicalis isolates resistant to AmB (64). Hence, we believe that comparing multiple ECN-R and ECN-S isolates can be helpful in limiting the detection of transcripts differentially expressed due to other isolate- or clade-specific properties. In line with this, some 362 genes were commonly differentially expressed among all three ECN-R and both ECN-S isolates. Of note, small transcriptomic alterations also occur among isolates with divergent antifungal susceptibility profiles (33). Also of note, a recent report detected almost 1,000 differentially expressed genes between azole-resistant and -susceptible C. auris isolates (65). This seems surprising as clade I as well as clade III isolates of C. auris display the lowest intraclade genetic diversities (13). Given that the syntenies and genomic arrangements are similar among the isolates investigated here, it is tempting to speculate that epigenetic factors contribute to such marked transcriptional alterations within a given clade. Thus, epigenetic signatures may be related to specific environmental stresses and pathogen “experiences” inherited by the next generation. Of note, the inheritance of stress-induced epigenetic marks is well established in mammals and plants (66). For instance, hereditary adaptive DNA methylation patterns in plants are harnessed to facilitate coevolution with new climatic conditions (67). Furthermore, the phytopathogenic fungus Cryphonectria parasitica shows epigenetic diversity among different haplotypes with distinctive expansion and invasion traits (68). Interestingly enough, genes mediating morphogenetic changes are enriched with heterogeneous DNA methylation patterns in C. albicans (69). Hence, hereditary epigenetic modifications, known as “epigenetic memory,” provide a swift adaptive-fitness advantage in response to environmental cues or host immune defenses and enable the phenotypic plasticity of clonal (sub)populations. These mechanisms are therefore considered key to the evolution of the onset and manifestation of infectious diseases (70, 71). Hence, assessing the dynamics of changing epigenetic landscapes among C. auris isolates coupled with transcriptional profiling is likely to yield valuable insights into the molecular and genetic mechanisms driving phenotypic diversification but also reveals the emergence of pathogenic virulence traits, including pan-antifungal resistance.

The rapid global spread and robust environmental persistence of C. auris as well as the increasing appearance of multi- and pan-antifungal-resistant isolates are of particular medical concern. While resistance to FCZ or AmB is frequently observed among clade I isolates, the frequency of ECN resistance remains low at the moment (11, 12, 35). As ECN stands out as the recommended first-line therapy against invasive candidiasis (72), an increase in ECN resistance among *Candida* spp. poses a serious threat to efficient antifungal therapies. Moreover, ECN resistance can develop quickly upon the onset of ECN treatment (73, 74). Mutations in *FKS* genes encoding β-glucan synthases are currently the major mechanisms underlying clinical ECN resistance. Drug exposure, such as with prophylactic or recurrent ECN treatment, further enhances the selection of resistance (39, 75). Of note, all of the investigated ECN-R isolates carry the *FKS1* S639F hot spot mutations, while the source patients had no history of ECN treatment. However, as C. auris can be transmitted from person to person by skin contact (76), the possibility remains that these isolates may originate from another patient with previous ECN exposure. Indeed, nearly clonal isolates have emerged from hospitals in north and south India (77) and can spread through local transmission (78).

Besides mutations in *FKS1*, additional regulators have been linked to increased CSP tolerance in other *Candida* spp., including Candida glabrata (39, 43, 74). For instance, the cell wall is intimately linked to ECN susceptibility. The genetic ablation of mannoproteins and other cell wall-related genes modulates CSP susceptibility in C. albicans and C. glabrata (43, 45). In line with this, CSP treatment readily triggers cell wall integrity signaling involving several pathways and transcription factors, thus leading to cell wall remodeling and stress adaptations (55, 79) such as increased chitin exposure (80, 81) via the activation of chitin synthesis. In line with this, C. auris elevates chitin levels in response to CSP treatment (82). In addition, a recent study observed elevated levels of exposed mannans in two C. auris isolates after 24 h of CSP exposure (55). Although the ECN-R isolates from our study did not display markedly increased chitin levels during standard laboratory growth, the mannan and β-glucan contents were substantially elevated in ECN-R strains. These distinct cell surface properties were further reflected in the altered adhesion potential and transcriptomic profiles of ECN-R isolates. Specifically, 16 genes annotated to the cellular component “cell wall” were commonly dysregulated in ECN-R C. auris isolates, with only 3 genes, *PGA6*, *PGA31*, and *XOG1*, being upregulated. Interestingly, *PGA31* expression was upregulated in CSP-treated C. albicans (83), and its deletion increased CSP susceptibility (45). Additionally, *PGA6* was also upregulated in other C. auris isolates treated with CSP for 24 h (55). However, single-gene deletions of *PGA6* did not affect CSP susceptibility in the isolates investigated here. Moreover, the loss of *PGA6* only moderately decreased cell adhesion by the ECN-R isolates. However, the effects varied between replicates. These results imply that the combinatorial loss of dysregulated cell surface genes may be required to affect the adhesion properties of ECN-R isolates substantially. In addition, the protein kinase Gin4, which controls septin function and regulation as well as cell wall integrity (84), was found to be upregulated in all ECN-R isolates. Similarly, *PGA31* was found to be upregulated during cell wall regeneration in response to protoplasting (85). The altered expression of otherwise stress-regulated genes under basal growth conditions may reflect differential baseline levels of cellular stress and environmental sensing. Besides *FKS* mutations conferring clinical ECN resistance, other mechanisms may contribute to the accumulation of additional genomic alterations mediating ECN resistance. Indeed, ECN resistance is a multistep developmental process consisting of stress adaptations such as cell wall integrity signaling (86). In line with this, an analysis tracking genomic alterations during CSP treatment in a human host revealed the occurrence of mutations that slightly decrease CSP susceptibility prior to the development of clinical ECN resistance via *FKS* mutations in C. glabrata (74). As the cell wall organization was altered in all three ECN-R isolates used in this study, we speculate that these cells might have initially displayed intrinsic tolerance to ECN prior to the acquisition of the *FKS1* S639F mutation. This would favor decreased fungal clearance in response to the initial ECN treatment. This manifested tolerance would then provide a window of opportunity to further facilitate adaptive mutations that drive ECN resistance. In addition, the ECN-R isolates displayed enhanced transcription of genes involved in mitochondrial gene expression (17 core set genes out of 51 genes annotated to mitochondrial gene expression). Interestingly, mitochondrial functions were recently implicated in ECN tolerance in C. glabrata (40) and were also enriched transcriptionally in CSP-treated C. auris (55). Hence, it is tempting to speculate that the detected core set of DEGs of ECN-R isolates, such as cell surface or mitochondrial genes, reflects phenotypic adaptive responses implicated in the development of ECN tolerance and, eventually, host interactions.

Antifungal drug resistance is most likely accompanied by trade-offs in fungal fitness in certain environments such as the absence of antifungal selection pressure (74, 87, 88). Indeed, the ECN-R isolates in this study showed decreased growth rates under host interaction conditions and ameliorated fungal recognition. However, the fungal killing of ECN-R isolates by BMDMs was not enhanced after 4 h of coculture. This indicates that although initial BMDM responses are decreased upon 717/P/14 challenge, the antifungal effector mechanisms of BMDMs still exceed the threshold for efficient fungal killing after prolonged interactions. Alternatively, ECN-R isolates may be more resistant to killing by BMDMs despite increased phagocytosis and ROS stress. The differential recognition of ECN-R strains versus the ECN-S 717/P/14 isolate may result from the decreased mannan abundance of ECN-S C. auris. Indeed, the mannan structure of the C. auris cell wall is considered key in mediating innate host responses (28). As ECN-R isolates have enhanced adhesion *in vitro*, these isolates may additionally show improved adhesion to host cells, which may increase fungal survival despite putative fitness defects. Thus, future studies should address the competitive fitness of C. auris strains displaying distinct phenotypic traits to further elucidate the factors mediating host interactions with different C. auris isolates.

Taken together, we identified a set of 362 genes commonly differentially regulated in three independent ECN-R isolates compared to two unrelated ECN-S C. auris isolates. This core gene set is enriched in, among others, cell wall-related genes, which are well represented in the phenotypic features of these isolates, as exemplified by the altered cell wall structure, adhesion, and host immune cell interactions of ECN-R isolates. Although we investigated only a few patient isolates, this work demonstrates in principle that phenotypic characteristics shared by distinct and unrelated patient isolates are well predictable by transcriptomic profiling. Hence, we suggest that the systematic profiling of C. auris transcriptomes and their integration with genomic data can facilitate the discovery of (clade-specific) transcriptional biomarkers of predictive value to assess host-fungal interactions, the propensity for antifungal resistance, and, possibly, therapeutic outcomes.

## MATERIALS AND METHODS

### Media and fungal growth conditions.

All strains used in this study are listed in Table S1 in the supplemental material. The clinical isolates of C. auris included 73% bloodstream isolates. The isolates were collected from the culture collection of the Medical Mycology Unit, Vallabhbhai Patel Chest Institute, University of Delhi, Delhi, India. *Candida* strains were routinely grown on YPD medium (1% yeast extract, 2% peptone, and 2% glucose [all from BD Biosciences]) at 30°C with shaking at 200 rpm. For solid medium, 2% Bacto agar (BD Biosciences) was added. Synthetic complete (SC) medium (1.7 g/L yeast nitrogen base without amino acids and ammonium sulfate [BD Biosciences], 5 g/L ammonium sulfate [Sigma-Aldrich], amino acid mix, and 2% glucose [both from BD Biosciences]) was prepared as previously described (89).

10.1128/mbio.00799-22.5 TABLE S1 Fungal strains used for antifungal susceptibility screening. Download Table S1, PDF file, 0.3 MB. Copyright © 2022 Jenull et al. 2022 Jenull et al. https://creativecommons.org/licenses/by/4.0/ This content is distributed under the terms of the Creative Commons Attribution 4.0 International license.

### Antifungal susceptibility screening on solid agar medium.

Antifungal susceptibility testing on solid medium was carried out essentially as described previously (33), using a robot instrument (RoToR HDA; Singer Ltd., Roadwater, UK). Briefly, C. auris clinical isolates were printed on YPD agar plates from cryostocks and incubated at 30°C for 3 days. Colony spots were inoculated in 200 μL liquid YPD medium in a 96-well plate using the robot instrument and grown overnight at 30°C with constant agitation (150 rpm) prior to spotting onto solid SC medium with or without antifungal drugs using the robot instrument. Growth inhibition by antifungal treatment was assessed by the colony size after incubation for 3 days at 30°C. The colony size was calculated using the R gitter package (https://github.com/omarwagih/gitter) and normalized to the colony size on SC medium without an antifungal drug (equal to the relative colony size). The lower the ratio of the colony size with the drug versus no drug, the more susceptible the isolate. The following antifungals were tested: fluconazole (FCZ; Discovery Fine Chemicals Ltd.) (64 μg/mL in dimethyl sulfoxide [DMSO] [Sigma-Aldrich]), itraconazole (ICZ; Discovery Fine Chemicals Ltd.) (0.15 μg/mL in DMSO), voriconazole (VCZ; Discovery Fine Chemicals Ltd.) (0.15 μg/mL in DMSO), amphotericin B (AmB; Santa Cruz Biotechnology) (3.0 μg/mL in DMSO), caspofungin (CSP; Merck) (0.40 μg/mL in distilled water [dH_2_O]), and 5-fluorocytosine (5-FC; Sigma-Aldrich) (10 μg/mL in dH_2_O).

### Growth inhibition assays.

Growth inhibition of C. auris isolates by antifungals, calcofluor white (Sigma-Aldrich), and antimycin A (Sigma-Aldrich) was assessed using an MIC assay in liquid YPD medium exactly as described previously (33). Optical density at 600 nm (OD_600_) readings were performed after 24 h of incubation at 30°C using a Victor Nivo plate reader (PerkinElmer). The percentage of growth represents the OD_600_ values in response to drug treatment relative to the OD_600_ readings of strains grown in YPD medium only. For drugs dissolved in DMSO, the corresponding DMSO concentration (2% final concentration) was included for untreated samples. In addition, MIC values of antifungals against C. auris isolates were assessed based on guidelines of Clinical and Laboratory Standards Institute (CLSI) M27-A3 protocols (90).

### Generation of gene deletion mutants.

C. auris gene deletion mutants were constructed using a fusion PCR strategy exactly as described previously (43). Briefly, roughly 500-bp flanking regions upstream and downstream of the C. auris target gene were amplified from genomic DNA (gDNA) extracted from the CBS10913 strain as described previously (33). The *NAT1* selection marker was amplified from the plasmid pTS50 (43). The PCR-amplified flanking regions and the *NAT1* selection marker were purified on a 1% agarose gel (PeqLab) and extracted using the GeneJET gel extraction kit (Thermo Scientific). Purified PCR products then served as the template for the fusion PCR to generate the final gene deletion cassette. The transformation of C. auris with the gene deletion cassette was carried out as reported previously (91). The correct genomic integration of the deletion construct and the loss of the target gene were verified by colony PCR (92). The oligonucleotides used in this study are listed in Table S2.

10.1128/mbio.00799-22.6 TABLE S2 Oligonucleotides used in this study. Download Table S2, PDF file, 0.2 MB. Copyright © 2022 Jenull et al. 2022 Jenull et al. https://creativecommons.org/licenses/by/4.0/ This content is distributed under the terms of the Creative Commons Attribution 4.0 International license.

### Transcriptional profiling using RNA sequencing.

For RNA sequencing (RNA-seq) analysis, C. auris cultures grown overnight were inoculated into YPD medium (initial OD_600_ of 0.1) and grown at 30°C for 4 h. Total RNA was purified using a GeneJET RNA purification kit (Thermo Scientific). The quality of RNA was assessed on a Bioanalyzer using the RNA6000 Nanochip (Agilent), mRNA was enriched using oligo(dT) beads (New England BioLabs [NEB]), and subsequently, double-stranded cDNA libraries were generated by using the NEBNext Ultra directional RNA library prep kit for Illumina (NEB) according to the manufacturer’s instructions. The qualified libraries were subjected to Illumina sequencing with 150-bp paired-end reads at the Novogene sequencing facility. Three biological replicates for each strain were sequenced.

Quality control of raw sequencing reads was done using fastQC v0.11.8 (93). TrueSeq (Illumina) adapters were trimmed using cutadapt v1.18 (https://cutadapt.readthedocs.io/en/stable/) (settings –interleaved -q 30), followed by read mapping onto the C. auris B8441 genome assembly (*Candida* Genome Database version s01-m01-r10 [http://www.candidagenome.org/]) using NextGenMap v0.5.5 (94) (settings -b -Q 30). Optical read duplicates were removed using Picard tools (Broad Institute [https://broadinstitute.github.io/picard/]) (settings MarkDuplicates REMOVE_SEQUENCING_DUPLICATES=true). Read counting was done using HTseq (95) in the union mode and the genomic annotation from C. auris B8841 (settings -f bam -r pos -t gene -i ID), and read coverage profiles were visualized using the Integrative Genomics Viewer (IGV) (96). Differential gene expression analysis was done using pairwise comparisons in edgeR (97). The false discovery rate (FDRs) represent *P* values adjusted for multiple testing using the Benjamini-Hochberg procedure (98). Normalized read counts were extracted using the edgeR cpm function and were used for principal-component analysis (PCA) using the prcomp function in R.

GO term enrichment analysis based on the C. albicans homologues was performed using the enrichGO function from the clusterProfiler package (99). Only GO categories with a *q* value of <0.05 were considered significant. Gene set enrichment analysis was done using the clusterProfiler gseGO function. C. albicans homologues were retrieved from the *Candida* Genome Database (http://www.candidagenome.org/). Venn diagrams were generated using the VennDiagram package in R (100). The RNA-seq analysis results are summarized in Table S3, all GO enrichment results are shown in Table S4, and data used to generate the heatmaps are shown in Table S5.

### Plastic adhesion assay.

C. auris isolates were grown to the logarithmic phase in YPD medium at 30°C and counted on a CASY counter (Roche). Cells were then diluted to 1 × 10^6^ cells/mL in YPD medium, and aliquots of 100 μL of this dilution were distributed into a well of a 96-well polystyrene plate (tissue culture treated; CytoOne). The plate was then incubated in a static incubator at 37°C for 4 h, and the adhered fungal biomass was subsequently quantified using crystal violet. Briefly, YPD medium was removed by inverting the plate and tapping it on paper towels. The wells were then washed three times with phosphate-buffered saline (PBS) and incubated with 100 μL methanol (Merck) for 15 min. The plates were left to dry overnight in a chemical safety cabinet, followed by staining with 100 μL 0.1% crystal violet for 5 min and three washes with dH_2_O. Crystal violet was dissolved from the stained biomass by adding 100 μL of 33% acetic acid and by plate shaking for 1 min at 800 rpm. The supernatants of dissolved crystal violet were transferred into fresh wells of a 96-well plate, and the absorbance at 590 nm was recorded using a Victor Nivo plate reader (PerkinElmer).

### Quantification of cell wall components.

Chitin, mannans, and exposed β-glucans were simultaneously quantified from C. auris isolates using flow cytometry as described previously, with modifications (101). Briefly, cells were precultured in YPD medium at 30°C with constant agitation for 4 to 5 h, followed by OD_600_ measurement. An aliquot of the preculture was then inoculated into fresh YPD medium to retrieve a culture at an OD_600_ of 1 to 2 after 15 to 17 h of further incubation at 30°C with constant agitation. The cells were then counted on a CASY counter (Roche), and 2 × 10^6^ cells were washed 3 times with fluorescence-activated cell sorter (FACS) buffer (1% fetal calf serum [FCS; Gibco], 0.5 mM EDTA, and 0.1% Tween 20 [both from Sigma-Aldrich] in PBS). To stain exposed β-glucans, cells were incubated with 5 ng/mL of Fc (human)–dectin-1 (AdipoGen) in FACS buffer on ice for 60 min. The cells were washed 3 times with FACS buffer and subsequently incubated with a 2.5-μg/mL final concentration of Alexa Fluor 488 anti-human IgG Fc antibody (BioLegend) on ice for 45 min, followed by 3 washes with FACS buffer and mannan staining using a 25-μg/mL final concentration of concanavalin A-Texas Red (Thermo Fisher) in FACS buffer at 30°C for 45 min. The cells were then washed again 3 times with FACS buffer, and chitin was stained with a 25-μg/mL final concentration of calcofluor white (Sigma-Aldrich) in FACS buffer. Samples were measured on an LSRFortessa cytometer (BD Biosciences), including unstained and singly stained controls.

The total β-glucan levels were assessed using a slightly modified aniline blue staining protocol described previously (102). Briefly, cells were grown as described above, put on ice, and washed twice with ice-cold PBS. Cells were then counted, diluted to 1 × 10^6^ cells/mL in a solution containing 1 M glycine (pH 9.5) and 0.005% aniline blue, and stained for 5 min. Unstained cells incubated for 5 min in 1 M glycine (pH 9.5) were included as controls. Samples were then subsequently measured on an LSRFortessa instrument (BD Biosciences).

Raw flow cytometry data were analyzed with FlowJo v7 (FlowJo software version 7.6.5), and the gating strategy is displayed in Fig. S3D.

### Coculture assays with primary macrophages.

Bone marrow-derived macrophages (BMDMs) were differentiated from the bone marrow of C57BL/6J mice essentially as described previously (103). For BMDM-C. auris cocultures, C. auris cells were cultured in YPD medium at 30°C with constant agitation to the logarithmic growth phase, washed 3 times with PBS, and counted on a CASY cell counter (Roche).

Fungal killing by BMDMs was analyzed as previously described (104). Briefly, 1 × 10^5^ BMDMs were seeded into a well of a 96-well plate (tissue culture treated; Starlab) 1 day prior to the assay. The next day, BMDMs were infected with 50 μL PBS containing 1 × 10^4^ C. auris cells, resulting in a multiplicity of infection (MOI) of 1:10 (fungi/BMDMs), and incubated for 4 h at 37°C with 5% CO_2_. Fungal cells were collected after the addition of 50 μL 4% SDS–PBS and well scraping with plastic tips. The contents of the scraped wells were transferred into 400 μL PBS, which were pooled with two PBS washes (200 μL per wash). Dilutions of the collected cells were plated onto YPD plates, and CFU were quantified after 48 h at 30°C. C. auris cells without BMDMs were cultured under otherwise identical conditions as the input control. The percentage of fungal killing represents the ratio of recovered CFU from C. auris cells cultured with BMDMs to CFU from C. auris single cultures (input control).

Phagocytosis assays with BMDMs were carried out as reported previously (105), with modifications. C. auris isolates were stained with 1 mg/mL FITC (Sigma-Aldrich) for 30 min at 30°C and washed 3 times with PBS prior to BMDM (2 × 10^5^ to 3 × 10^5^ BMDMs per well of a 24-well plate [Starlab]) infection at an MOI of 2:1 (fungi/BMDMs) for 45 and 120 min at 37°C with 5% CO_2_. In parallel, a BMDM-C. auris coculture was kept at 4°C as a negative control. After the incubation time, the plates were immediately put on ice, and BMDMs were washed 3 times with cold PBS. Extracellular FITC fluorescence was quenched with 200 μL 0.4% trypan blue (Sigma-Aldrich) for 15 min at 4°C, followed by 3 washes with cold PBS. BMDMs were then treated with 250 μL trypsin (Sigma-Aldrich) for 5 min at 37°C and harvested in 750 μL Dulbecco’s modified Eagle’s medium (DMEM)–10% heat-inactivated FCS (hiFCS) by pipetting. BMDMs were pelleted at 300 × *g* at 4°C for 4 min and resuspended in 300 μL FACS buffer (PBS plus 0.1% bovine serum albumin [BSA] [both from Sigma-Aldrich]). Samples were measured on an LSRFortessa cytometer (BD Biosciences), including BMDMs without FITC-labeled C. auris and C. auris singly stained controls. Raw flow cytometry data were analyzed with FlowJo v7 (FlowJo software version 7.6.5).

Reactive oxygen species (ROS) responses in BMDMs cocultured with C. auris were assessed using a luminol-based assay essentially as described previously (106). Briefly, 4 × 10^4^ BMDMs were seeded into a well of a 96-well plate (tissue culture treated, white walled; Thermo Scientific) the day prior to the assays. BMDMs were then washed twice with prewarmed PBS, followed by the addition of 100 μL prewarmed Hanks’ balanced salt solution (HBSS) with Mg^2+^ and Ca^2+^ (Gibco). C. auris cells were resuspended in HBSS with Mg^2+^ and Ca^2+^ and adjusted to 4 × 10^6^ fungal cells per mL. Luminol and horseradish peroxidase (HRP) type VI (both from Sigma) were diluted in HBSS with Mg^2+^ and Ca^2+^ to final concentrations of 200 μM and 16 U, respectively, and 50 μL of this mix was added to the BMDMs, followed by the addition of 50 μL of the adjusted C. auris cell suspension, resulting in an MOI of 5:1 (fungi/BMDMs). Chemiluminescence was recorded in real time at 2.5-min intervals at 30°C using a Victor Nivo plate reader (PerkinElmer). Raw relative luciferase units (RLU) were blanked with values from BMDM single cultures and are presented as RLU per minute over time or as total RLU for the indicated time span.

### Fungal growth in BMDM medium.

C. auris cells were grown in YPD medium overnight at 30°C with constant agitation. Cultures were washed twice in dH_2_O prior to reinoculation into BMDM medium (L-conditioned DMEM [high glucose without pyruvate] and 10% heat-inactivated FCS) “to” (DMEM [high glucose without pyruvate] containing 15% L929-conditioned cell supernatant and 10% heat-inactivated FCS [L-conditioned DMEM]).

### Statistical analysis.

Statistical significance was assessed using GraphPad Prism 9 (GraphPad Software, Inc.) or RStudio (107). The number of biological replicates is stated in each figure legend. Error bars represent the standard deviations (SD) of the means. Unless otherwise stated, two-sample comparisons were analyzed using unpaired two-sided Student’s *t* tests. Multigroup comparisons were assessed using one-way analysis of variance (ANOVA) with Tukey’s multiple-comparison correction. IC_50_ values were calculated using nonlinear regression (four-parameter dose-response curve) in GraphPad Prism 9 (GraphPad Software, Inc.) (*, *P* < 0.05; **, *P* < 0.01; ***, *P* < 0.001; ns, not statistically significant).

### Data availability.

RNA-seq data sets have been deposited at the Gene Expression Omnibus (GEO) (accession number GSE198410). Scripts used for the primary RNA-seq bioinformatics workflow are freely available on GitHub (https://github.com/tschemic/RNAseq_analysis_Cauris).
