# Supplementary figures and images for: Transcriptomics and Phenotyping Define Genetic Signatures Associated with Echinocandin Resistance in Candida auris
Source: mBio. 2022 Aug 15;13(4):e00799-22. doi: 10.1128/mbio.00799-22 (PMC9426441; doi:10.1128/mbio.00799-22)

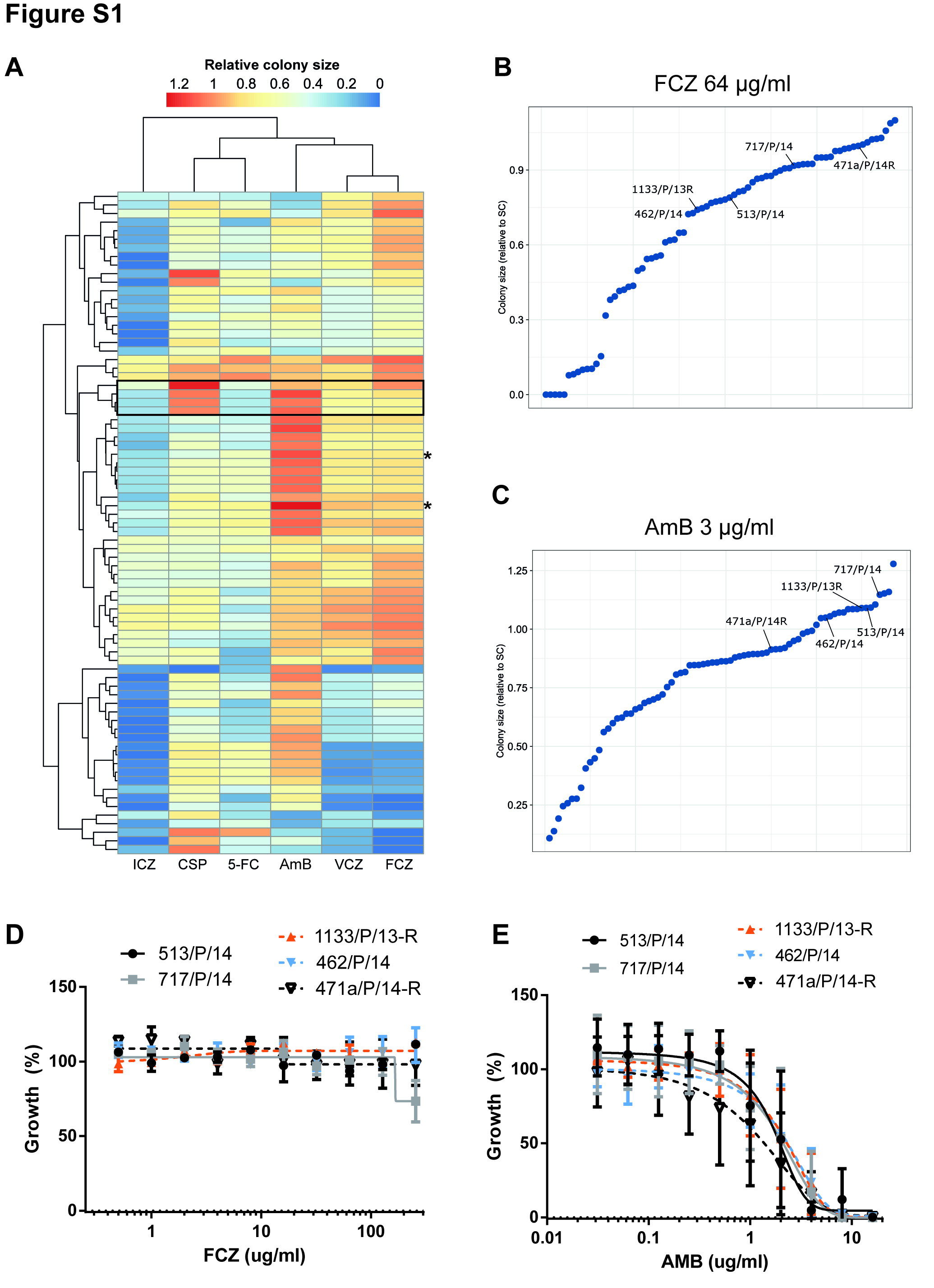

Supplement: FIG S1 [file mbio.00799-22-s0004.tif]

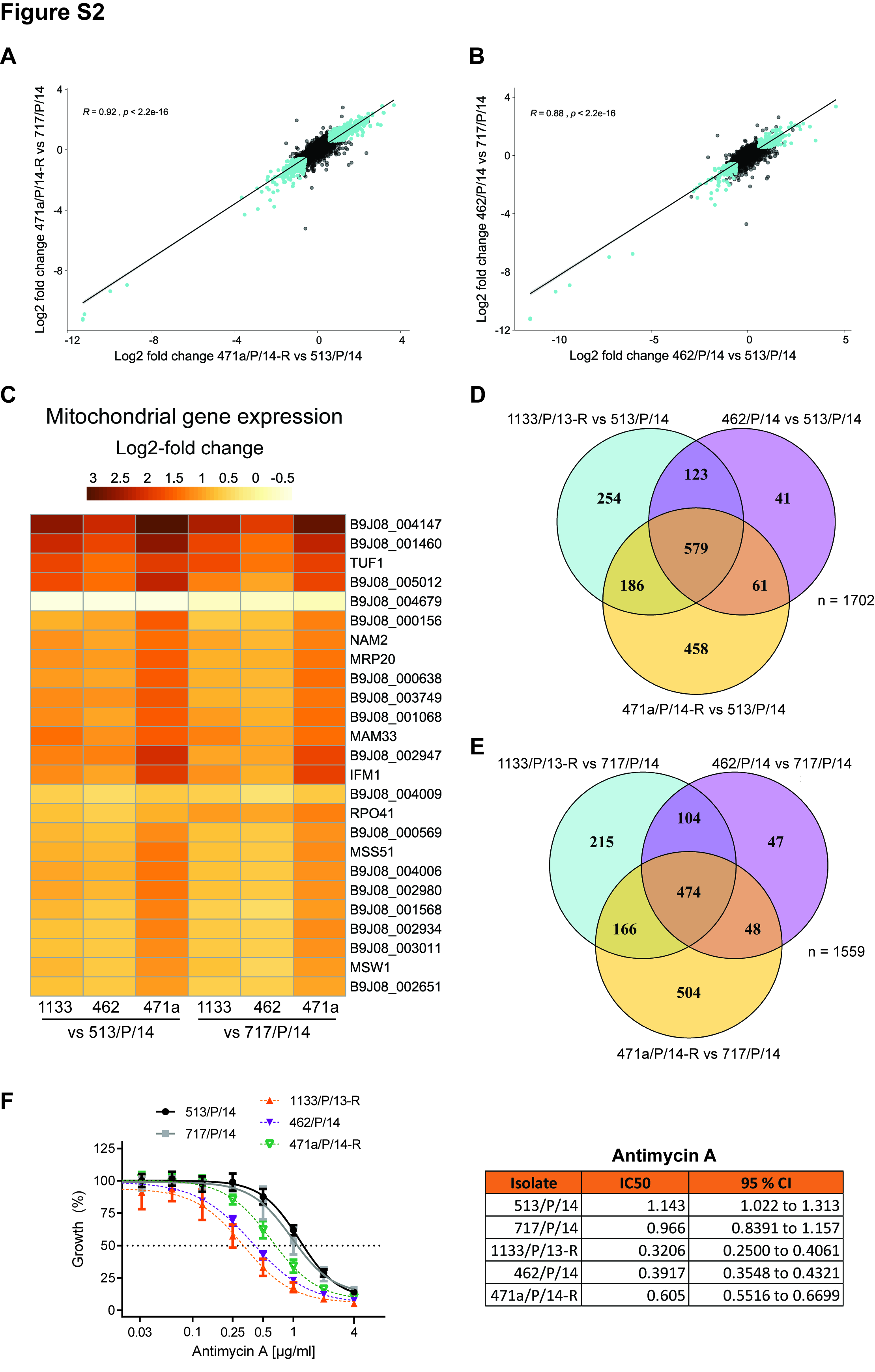

Supplement: FIG S2 [file mbio.00799-22-s0005.tif]

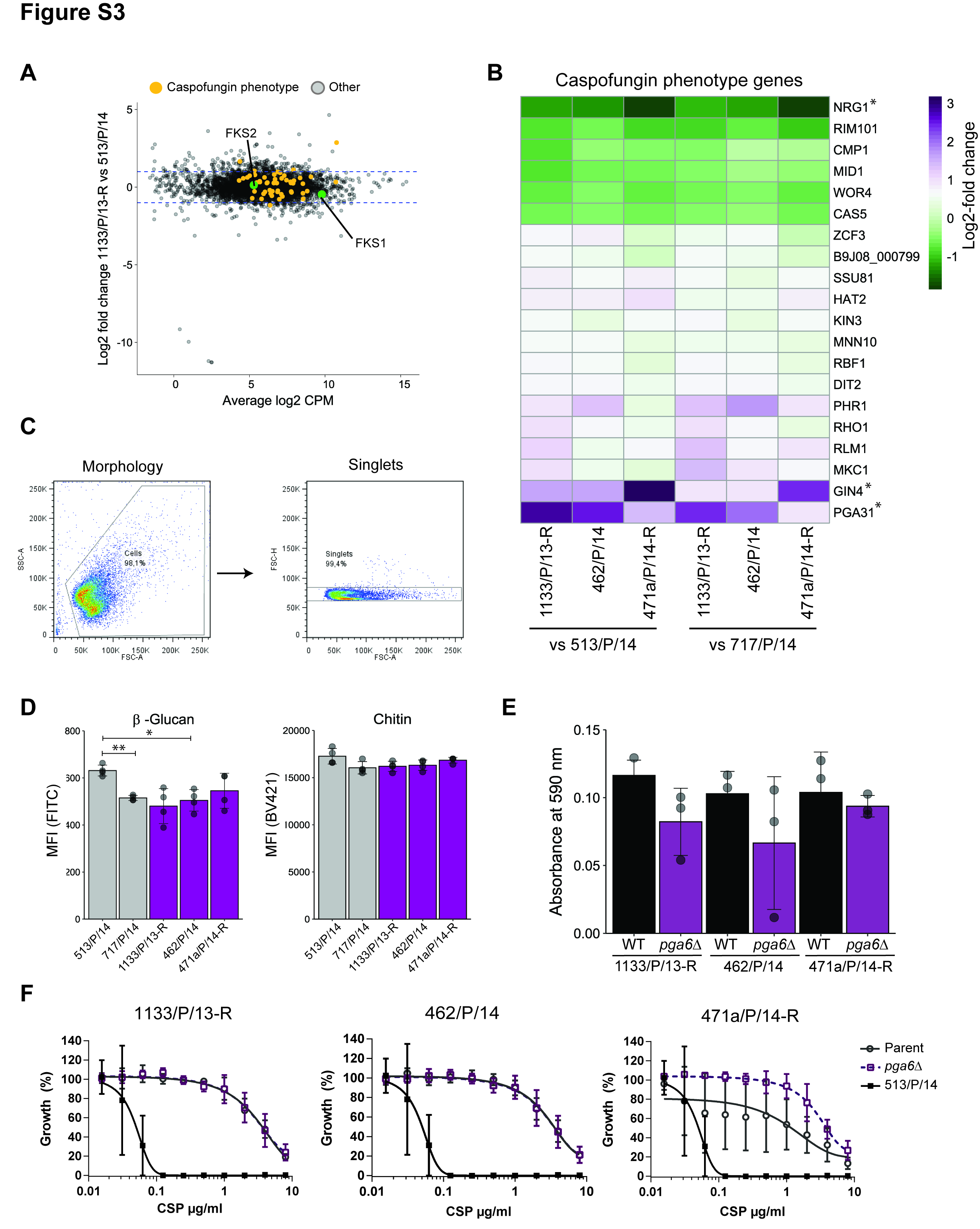

Supplement: FIG S3 [file mbio.00799-22-s0006.tif]

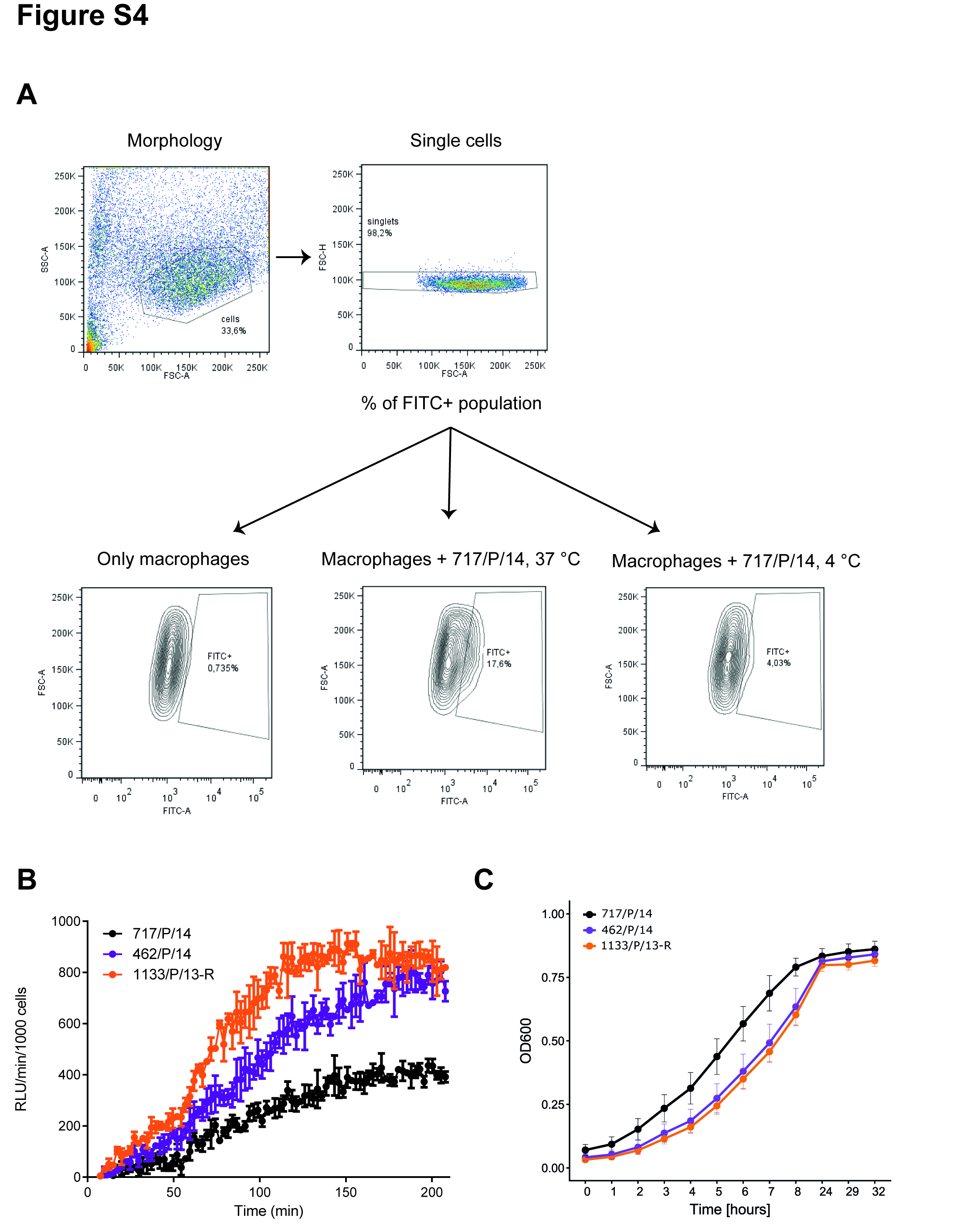

Supplement: FIG S4 [file mbio.00799-22-s0007.tif]
