# Supplementary material for: Transcriptomics and Phenotyping Define Genetic Signatures Associated with Echinocandin Resistance in Candida auris
Source: mBio. 2022 Aug 15;13(4):e00799-22. doi: 10.1128/mbio.00799-22 (PMC9426441; doi:10.1128/mbio.00799-22)
Supplement: TABLE S1 [file mbio.00799-22-s0008.pdf]

Table S1. Fungal strains used for antifungal susceptibility screening

Strain ID	Type/Specimen	Source
Candida albicans SC5314	Clinical Isolate	Gillum, A. M., Tsay, E. Y. H., & Kirsch, D. R. (1984), MGG Molecular & General Genetics, 198(1), 179–182. https://doi.org/10.1007/BF00328721
Candida glabrata ATCC2001	Clinical Isolate	Dujon, B et al., (2004), Nature, 430(6995), 35–44. https://doi.org/10.1038/nature02579
Candida glabrata HTL	Auxotrophic *Candida glabrata* control strain	Schwarzmüller et al., (2014), PLoS Pathogens, 10(6). https://doi.org/10.1371/journal.ppat.1004211
CBS 5149 (C. haemulonii)	Type strain	Neeraj Chauhan
cdr1::delta-40154	*CDR1* gene deletion strain in *C. auris* 470154	Jenull et al., (2021), Frontiers in Cellular and Infection Microbiology, 11, 662563. https://doi.org/10.3389/fcimb.2021.662563
cdr1::detlta-CBS10913	*CDR1* gene deletion strain in *C. auris* CBS10913	Jenull et al., (2021), Frontiers in Cellular and Infection Microbiology, 11, 662563. https://doi.org/10.3389/fcimb.2021.662563
HTL Δ*slt2*	*SLT2* gene deletion control strain in HTL	Schwarzmüller et al., (2014), PLoS Pathogens, 10(6). https://doi.org/10.1371/journal.ppat.1004211
HTL Δ*ypk1*	*YPK1* Gene deletion control strain in HTL	Schwarzmüller et al., (2014), PLoS Pathogens, 10(6). https://doi.org/10.1371/journal.ppat.1004211
VPCI_1030/P/17	Clinical Isolate/Blood	Medical Mycology Unit, VPCI
VPCI_1069/P/17	Clinical Isolate/Blood	Medical Mycology Unit, VPCI
VPCI_107/P/14	Clinical Isolate/BAL	Medical Mycology Unit, VPCI
VPCI_1098/P/17	Clinical Isolate/Blood	Medical Mycology Unit, VPCI
VPCI_1117/P/17	Clinical Isolate/Blood	Medical Mycology Unit, VPCI
VPCI_1121/P/17	Clinical Isolate/Blood	Medical Mycology Unit, VPCI
VPCI_1131/P/13	Clinical Isolate/Tissue	Medical Mycology Unit, VPCI
VPCI_1133/P/13	Clinical Isolate/Blood	Medical Mycology Unit, VPCI
VPCI_1133/P/13-R	Clinical Isolate/Blood	Medical Mycology Unit, VPCI
VPCI_1184/P/15	Clinical Isolate/Blood	Medical Mycology Unit, VPCI
VPCI_1237/P/15	Clinical Isolate/Blood	Medical Mycology Unit, VPCI
VPCI_1244/P/15	Clinical Isolate/Blood	Medical Mycology Unit, VPCI
VPCI_1273/P/16	Clinical Isolate/Blood	Medical Mycology Unit, VPCI
VPCI_1361/P/15	Clinical Isolate/Tissue	Medical Mycology Unit, VPCI
VPCI_1362/P/15	Clinical Isolate/Urine	Medical Mycology Unit, VPCI
VPCI_1583/P/17	Clinical Isolate/Groin	Medical Mycology Unit, VPCI
VPCI_1596/P/17	Clinical Isolate/Blood	Medical Mycology Unit, VPCI
VPCI_1676/P/18	Clinical Isolate/Blood	Medical Mycology Unit, VPCI

VPCI_1781/P/18	Clinical Isolate/Urine	Medical Mycology Unit, VPCI
VPCI_1783/P/16	Clinical Isolate/ET	Medical Mycology Unit, VPCI
VPCI_1794/P/18	Clinical Isolate/Blood	Medical Mycology Unit, VPCI
VPCI_1816/P/16	Clinical Isolate/Blood	Medical Mycology Unit, VPCI
VPCI_1894/P/18	Clinical Isolate/Blood	Medical Mycology Unit, VPCI
VPCI_1897/P/18	Clinical Isolate/Blood	Medical Mycology Unit, VPCI
VPCI_1991/P/16	Clinical Isolate/Urine	Medical Mycology Unit, VPCI
VPCI_205/P/19	Clinical Isolate/Vaginal Swab	Medical Mycology Unit, VPCI
VPCI_213/P/15	Clinical Isolate/Blood	Medical Mycology Unit, VPCI
VPCI_215/P/15	Clinical Isolate/Tissue	Medical Mycology Unit, VPCI
VPCI_2431/P/16	Clinical Isolate/Blood	Medical Mycology Unit, VPCI
VPCI_2447/P/16	Clinical Isolate/Groin	Medical Mycology Unit, VPCI
VPCI_248/P/14	Clinical Isolate/Blood	Medical Mycology Unit, VPCI
VPCI_249/P/14	Clinical Isolate/Blood	Medical Mycology Unit, VPCI
VPCI_260/P/14	Clinical Isolate/Blood	Medical Mycology Unit, VPCI
VPCI_265/P/14	Clinical Isolate/Blood	Medical Mycology Unit, VPCI
VPCI_320/P/19	Clinical Isolate/Blood	Medical Mycology Unit, VPCI
VPCI_343/P/19	Clinical Isolate/Blood	Medical Mycology Unit, VPCI
VPCI_431/P/15	Clinical Isolate/Blood	Medical Mycology Unit, VPCI
VPCI_462/P/14	Clinical Isolate/Blood	Medical Mycology Unit, VPCI
VPCI_471/P/13	Clinical Isolate/BAL	Medical Mycology Unit, VPCI
VPCI_471a/P/14-R	Clinical Isolate/Blood	Medical Mycology Unit, VPCI
VPCI_478/P/13	Clinical Isolate/Pus	Medical Mycology Unit, VPCI
VPCI_482/P/13	Clinical Isolate/Tissue	Medical Mycology Unit, VPCI
VPCI_507/P/14	Clinical Isolate/Urine	Medical Mycology Unit, VPCI
VPCI_510/P/14	Clinical Isolate/Blood	Medical Mycology Unit, VPCI
VPCI_513/P/14	Clinical Isolate/Blood	Medical Mycology Unit, VPCI
VPCI_520/P/15	Clinical Isolate/Blood	Medical Mycology Unit, VPCI
VPCI_594/P/16	Clinical Isolate/Blood	Medical Mycology Unit, VPCI
VPCI_601/P/16	Clinical Isolate/Urine	Medical Mycology Unit, VPCI
VPCI_614/P/15	Clinical Isolate/Blood	Medical Mycology Unit, VPCI
VPCI_669/P/12	Clinical Isolate/Blood	Medical Mycology Unit, VPCI
VPCI_676/P/12	Clinical Isolate/Blood	Medical Mycology Unit, VPCI
VPCI_682/P/15	Clinical Isolate/Blood	Medical Mycology Unit, VPCI
VPCI_635/P/19	Clinical Isolate/Blood	Medical Mycology Unit, VPCI
VPCI_701/P/19	Clinical Isolate/Blood	Medical Mycology Unit, VPCI
VPCI_712/P/12	Clinical Isolate/Blood	Medical Mycology Unit, VPCI
VPCI_714/P/16	Clinical Isolate/Blood	Medical Mycology Unit, VPCI
VPCI_717/P/14	Clinical Isolate/Tissue	Medical Mycology Unit, VPCI
VPCI_718/P/14	Clinical Isolate/Blood	Medical Mycology Unit, VPCI
VPCI_737/P/16	Clinical Isolate/Blood	Medical Mycology Unit, VPCI
VPCI_853/P/18	Clinical Isolate/Blood	Medical Mycology Unit, VPCI
VPCI_900/P/15	Clinical Isolate/Blood	Medical Mycology Unit, VPCI

VPCI_940/P/16	Clinical Isolate/Blood	Medical Mycology Unit, VPCI
VPCI_960/P/17	Clinical Isolate/Blood	Medical Mycology Unit, VPCI
VPCI_974/P/17	Clinical Isolate/ Blood	Medical Mycology Unit, VPCI
