# Supplementary material for: Transcriptomics and Phenotyping Define Genetic Signatures Associated with Echinocandin Resistance in Candida auris
Source: mBio. 2022 Aug 15;13(4):e00799-22. doi: 10.1128/mbio.00799-22 (PMC9426441; doi:10.1128/mbio.00799-22)
Supplement: TABLE S2 [file mbio.00799-22-s0009.pdf]

Table S2. Oligos used in this study.

Name	Sequence (5'->3')	Purpose
55_CaurPGA6	CAGAGAGAGTAACGAGCAAGG	Forwrad primer amplifying the region upstream of PGA6 (B9J08_001366)
53_CaurPGA6	cacggcgcgcctagcagcggCGAATGTATAAGTTGGGATAAGTGG	Reverse primer amplifying the region upstream of PGA6 (B9J08_001366)
35_CaurPGA6	gtcagcggccgcatccctgcGGATAAGTTTAATTCAACACTGCC	Forwrad primer amplifying the region downstream of PGA6 (B9J08_001366)
33_CaurPGA6	CACTTAGAAGATGTTGCTGCTG	Reverse primer amplifying the region downstream of PGA6 (B9J08_001366)
5C_CaurPGA6	CTAGGCACGGATTCACAAAGG	5' integration check of the PGA6 deletion cassette
3C_CaurPGA6	CCAGCACTTGTTGAAGTTCC	3' integration check of the PGA6 deletion cassette
CaurPGA6_LOG_fwd	CAACAAGCACACCACCGAG	Forward primer binding within the ORF of PGA6 - loss of gene check
CaurPGA6_LOG_rev	GTGTGCTCGACACCAACAGAG	Reverse primer binding within the ORF of PGA6 - loss of gene check
hk2	CGTCAAGACTGTCAAGGAGGG	Integration check of the deletion cassette binding within the *NAT1* cassette
hk3	CATCATCTGCCCAGATGCGAAG	Integration check of the deletion cassette binding within the *NAT1* cassette
5M_A4	ccgctgctaggcgcgccgtgATCGAACGTGTGTCAAACGCcgtacgctgcaggtcgac	Amplification of the NAT1 marker from pTS50 containing barcode A4 (Schwarzmüller et al, (2014), PLoS Pathogens, 10(6). https://doi.org/10.1371/journal.ppat.1004211). Used for *PGA6* deletion mutant.
3M_A4	gcagggatgcggccgctgacATAGCCGACTAACGAGCGTCctacgagaccgacaccg	Amplification of the NAT1 marker from pTS50 containing barcode A4 (Schwarzmüller et al, (2014), PLoS Pathogens, 10(6). https://doi.org/10.1371/journal.ppat.1004211); Used for *PGA6* deletion mutant.
